# Editorial for Special Issue: Advances in Microfluidic Devices for Cell Handling and Analysis

**DOI:** 10.3390/mi8060184

**Published:** 2017-06-09

**Authors:** Abel Martin Gonzalez Oliva

**Affiliations:** Instituto de Tecnologia Química e Biológica Antonio Xavier, Universidade Nova de Lisboa, Avenida da Republica, 2780-157 Oeiras, Portugal; oliva@itqb.unl.pt; Tel.: +351-21-446-9427

Microfluidics is a technology that is expanding rapidly in many areas of research, especially in the biological areas of cell handling and analysis. The manipulation of fluids at the submillimeter scale in coordination with improved sensory and monitoring methods has increased the capacity for diagnostics and biology research dramatically. Certain properties of microfluidic technologies, such as precise control of fluids in an assay and manipulation of particles at a microscale, made them a powerful tool for cell studies. Measurements at the level of a single cell are one of the most challenging and informative in systems biology. Advances in medicine, biotechnology, and agriculture—among other areas—are possible by these new approaches at an almost individual cell-scale.

Here, we present a Special Issue entitled “Advances in Microfluidic Devices for Cell Handling and Analysis”, addressing recent advances in the instrumentation, fabrication, and characterization of microfluidic devices for this purpose. The selected works deal with cell handling, cell trapping, cell characterization by different methods, and includes nine original papers, two of them as reviews.

The special issue includes some papers dealing directly with cell handling and manipulation, as the work of Huang et al. [1] that developed an integrated single cell trap platform with a novel configuration of electrodes for cell 3D rotation manipulation or the original work of Mizoue et al. [2]. Jo and coauthors have been developing a simple and robust single cell sorting platform based on a magnetophoretic method using monodisperse magnetic nanoparticles (MNPs) and droplet microfluidics with >94% purity [3]. An interesting technique is presented by Katrin Rosenthal et al. [4] towards a continuous microreactor for the isolation and analysis of a single microbial cell.

This special issue includes some works of cell monitoring exploring different approaches as in the work of Pereira et al. [5] that developed a novel microfluidic platform for multifactorial analysis integrating four label-free detection methods: electrical impedance, refractometry, optical absorption, and fluorescence or the work of Li and Yuan [6], whom designed and developed vertical electrodes for impedance analysis of cells in a microchannel.

An original paper of by Joana Calejo et al. [7] describes a particulate analog for blood, that reproduces erythrocytes’ properties, overcoming the problem of manipulation of blood by microdevices for experimental hemodynamic studies and avoiding the difficulties associated with the manipulation of real samples.

Two reviews complete the special issue, one dealing with micro-controlled cell environment and monitoring [8] and other discussing recent developments of cell monitoring and manipulation systems onto glass substrate chips [9].

I am sure this will be of high interest for the researchers and engineers working in the field of microfluidic cell manipulation as well as for readers of other areas of research in which such microsystems and technologies are gaining more attraction for its exceptional possibilities. Enjoy it!

## References

[1] Huang L., Tu L., Zeng X., Mi L., Li X., Wang W. (2016). Study of a Microfluidic Chip Integrating Single Cell Trap and 3D Stable Rotation Manipulation. Micromachines.

[2] Mizoue K., Phan M.H., Tsai C.-H.D., Kaneko M., Kang J., Chung W.K. (2016). Gravity-Based Precise Cell Manipulation System Enhanced by In-Phase Mechanism. Micromachines.

[3] Jo Y., Shen F., Hahn Y.K., Park J.-H., Park J.-K. (2016). Magnetophoretic Sorting of Single Cell-Containing Microdroplets. Micromachines.

[4] Rosenthal K., Falke F., Frick O., Dusny C., Schmid A. (2015). An Inert Continuous Microreactor for the Isolation and Analysis of a Single Microbial Cell. Micromachines.

[5] Pereira F.M., Bernacka-Wojcik I., Ribeiro R.S.R., Lobato M.T., Fortunato E., Martins R., Igreja R., Jorge P.A.S., Águas H., Oliva A.M.G. (2016). Hybrid Microfluidic Platform for Multifactorial Analysis Based on Electrical Impedance, Refractometry, Optical Absorption and Fluorescence. Micromachines.

[6] Li Q., Yuan Y.J. (2016). Application of Vertical Electrodes in Microfluidic Channels for Impedance Analysis. Micromachines.

[7] Calejo J., Pinho D., Galindo-Rosales F.J., Lima R., Campo-Deaño L. (2016). Particulate Blood Analogues Reproducing the Erythrocytes Cell-Free Layer in a Microfluidic Device Containing a Hyperbolic Contraction. Micromachines.

[8] Azuaje-Hualde E., García-Hernando M., Etxebarria-Elezgarai J., De Pancorbo M.M., Benito-Lopez F., Basabe-Desmonts L. (2017). Microtechnologies for Cell Microenvironment Control and Monitoring. Micromachines.

[9] Buehler S.M., Stubbe M., Bonk S.M., Nissen M., Titipornpun K., Klinkenberg E.-D., Baumann W., Gimsa J. (2016). Cell Monitoring and Manipulation Systems (CMMSs) based on Glass Cell-Culture Chips (GC^3^s). Micromachines.

